# Supplementary material for: Origin of the unusually strong and selective binding of vanadium by polyamidoximes in seawater
Source: Nat Commun. 2017 Nov 16;8:1560. doi: 10.1038/s41467-017-01443-1 (PMC5691157; doi:10.1038/s41467-017-01443-1)
Supplement: Supplementary file 1 — Supplementary Information [file 41467_2017_1443_MOESM1_ESM.pdf]

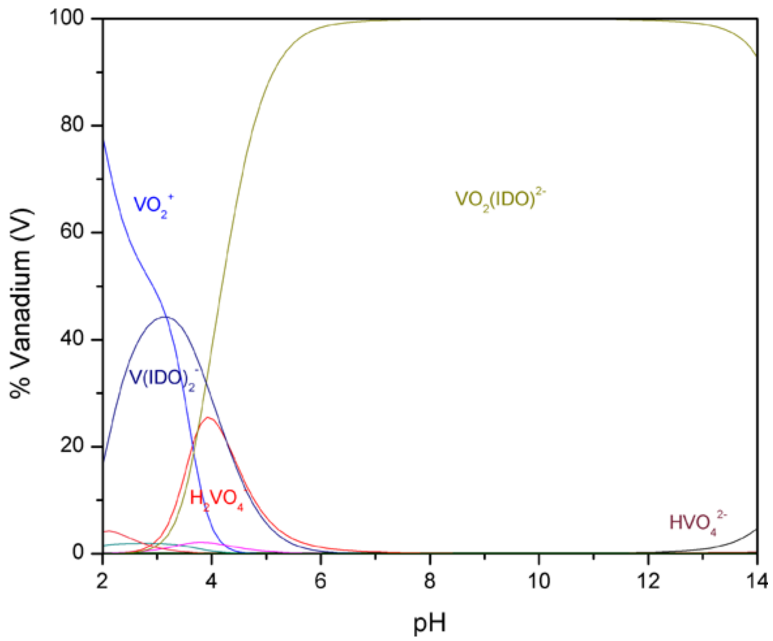

Supplementary Figure 1. Species distribution diagram generated using theoretically predicted *log β*^{theor} values for 1.0 mM [H_3IDO] and 1.0 mM [V] ([H_3IDO]/[V]= 1/1 ratio) as a function of pH.

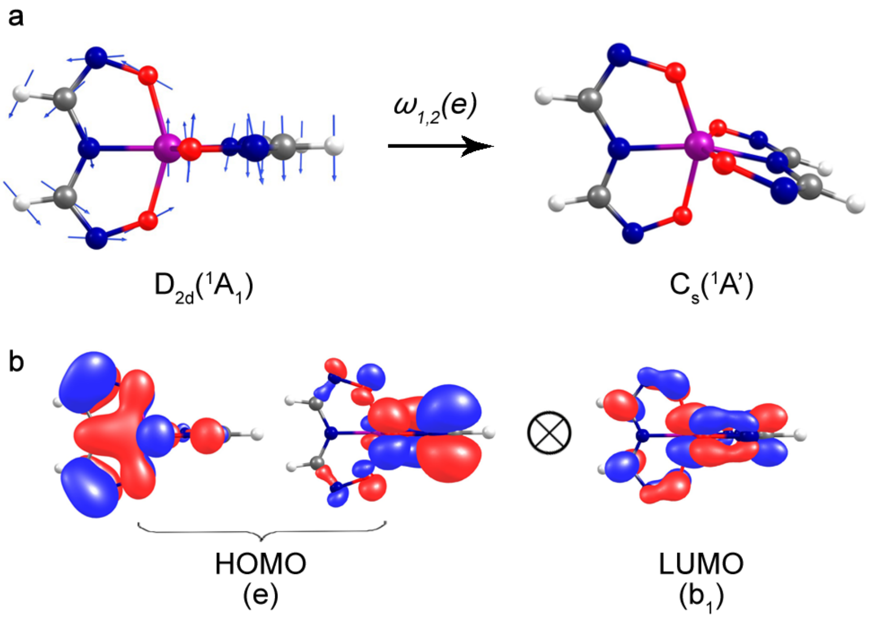

Supplementary Figure 2. Pseudo-Jahn-Teller (PJT) origin of distortions in the $[V(IDO)_2]^-$ complex. **a** Distortion of the D_{2d} symmetric $[V(IDO')_2]^-$ complex along the *e* imaginary mode; vibrational vector for the PJT *e* mode is depicted in blue. **b** Interaction of the pairs of occupied and unoccupied molecular orbitals of the D_{2d} ($^1A_1$) structure of $[V(IDO')_2]^-$ responsible for the PJT effect: interactions cause distortion toward the C_s ($^1A'$) structure upon following the doubly degenerate $\omega_{1,2}$ (*e*) imaginary frequency mode.

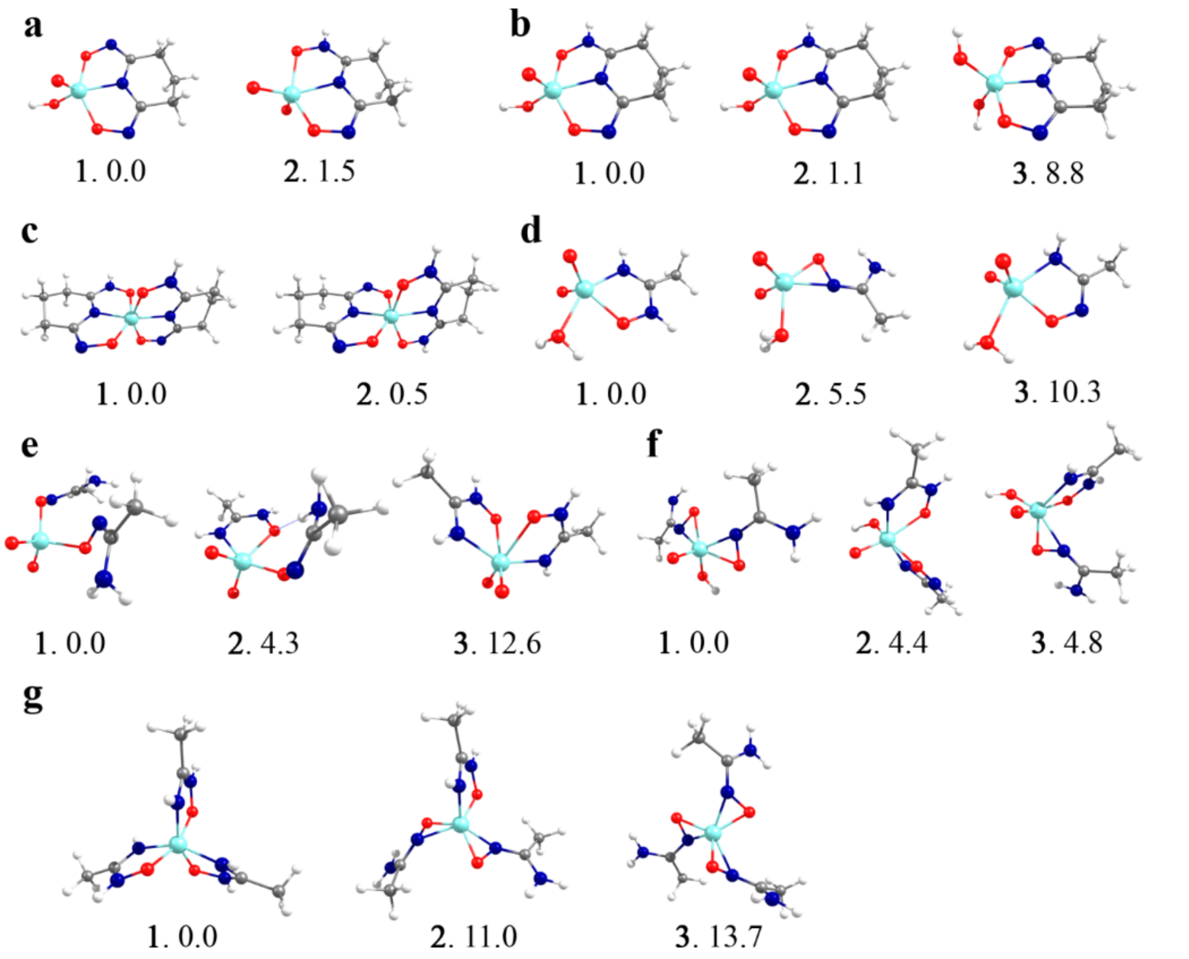

Supplementary Figure 3. Optimized structures (M06/SSC/6-311++G**) for different stoichiometries and their relative Gibbs free energies (kcal/mol) in aqueous solution. **a** $[VO_2(HIDO)]^-$, **b** $[VO_2(H_2IDO)]$, **c** $[V(HIDO)_2]^+$, **d** $[VO_2(AO)]$, **e** $[VO_2(AO)_2]^-$, **f** $[VO(OH)(AO)_2]$, **g** $[V(AO)_3]^{2+}$. Color legend: V(V), turquoise; O, red; N, blue; C, grey; H, white.

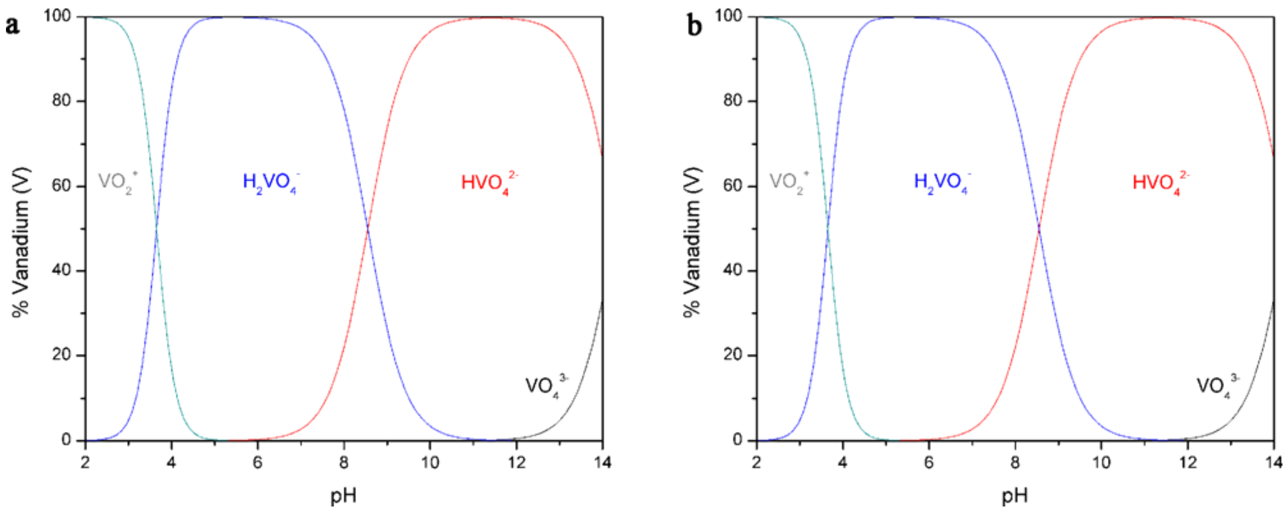

Supplementary Figure 4. Species distribution diagrams generated using theoretically predicted *log* β^{theor} values for **a** 1 mM [HAO] and 1 mM [V] ([HAO]/[V]= 1/1 ratio) and **b** 15 mM [HAO] and 1 mM [V] ([HAO]/[V]= 15/1 ratio) as a function of pH.

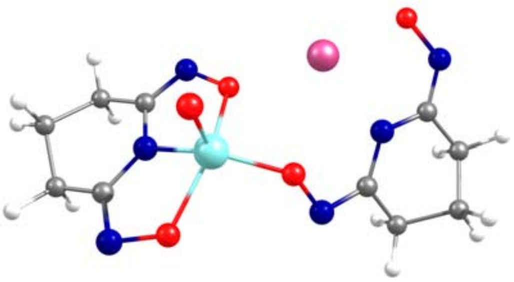

Supplementary Figure 5. Optimized structure (M06/SSC/6-311++G**) of the $[VO(IDO)_2]^{3-}$ $[Na^+]$ complex. Color scheme: O, red; N, blue; C, grey; H, white; V, turquoise; Na, pink.

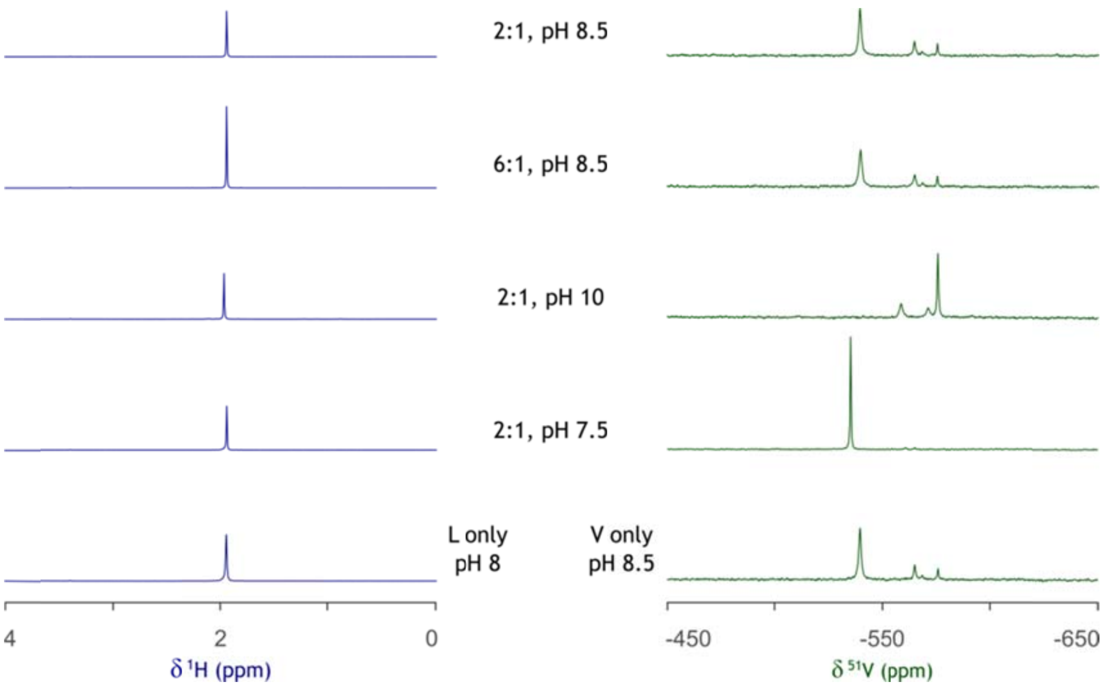

Supplementary Figure 6. ^{1}H and ^{51}V NMR spectra of acetamidoxime (HAO) with vanadate.

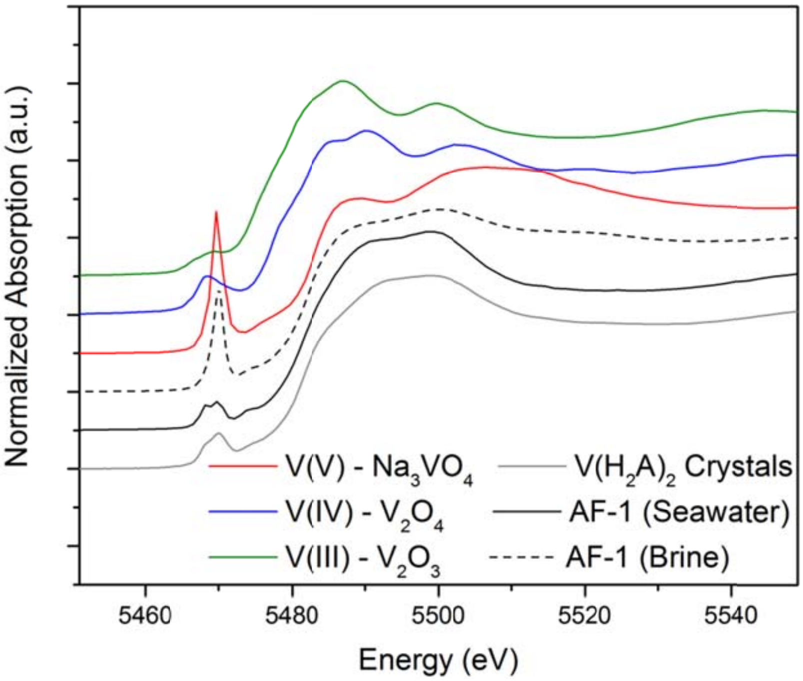

Supplementary Figure 7. X-ray Absorption Near Edge Spectra (XANES) for representative brine- and seawater-contacted adsorbents, Na[V(IDO)$_2$] single crystal, and mineral standards of Na_3VO_4, V_2O_4, and V_2O_3 displaying E_0 values for oxidation states of V, IV, and III, respectively. The absorption edge of the brine- and seawater-contacted adsorbents possess an E_0 value consistent with Na_3VO_4 (5482 eV), supporting an oxidation state of V(V). E_0 values for V(IV) and V(III) are 5478 eV and 5475 eV, respectively.

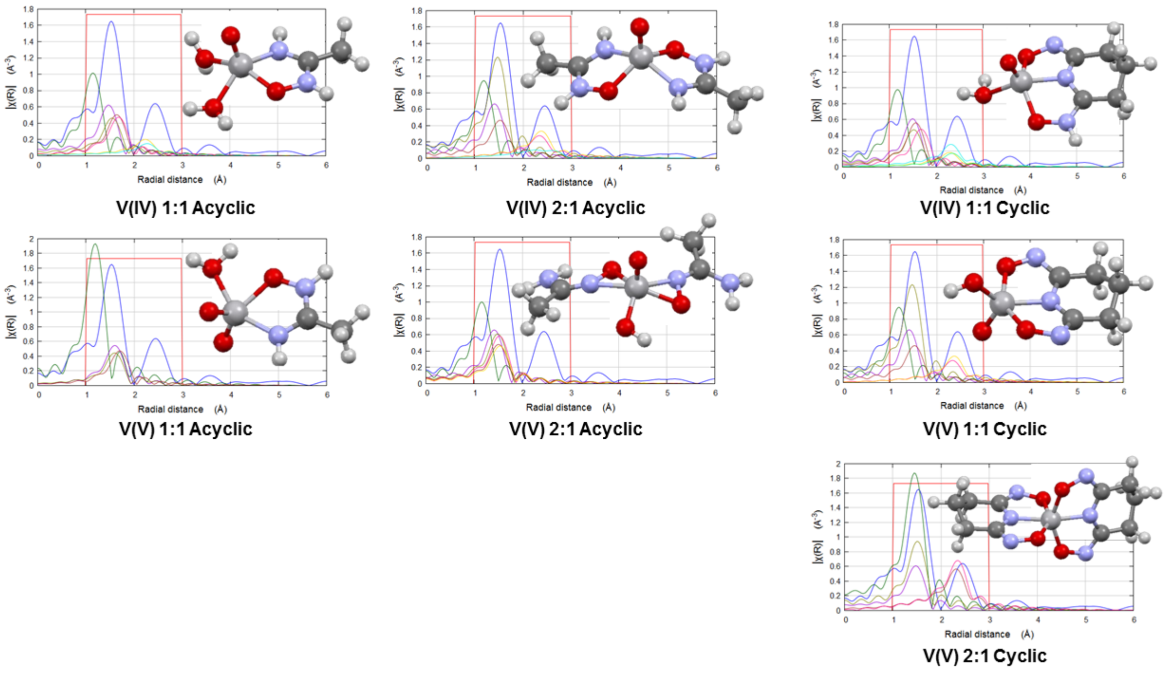

Supplementary Figure 8. Representative EXAFS spectrum for seawater-contacted adsorbents and simulated scattering paths for computationally proposed vanadium binding modes. The top row shows geometrically optimized vanadium(IV) binding modes while the bottom four plots are vanadium(V) binding modes. The left column displays 1:1 acyclic binding modes, the centre column displays 2:1 acyclic binding modes, and the right column displays cyclic binding modes. Experimental EXAFS data are displayed in blue with the simulated scattering paths superpositioned in different colors. The red box displays the fit window. From this qualitative comparison, it is apparent that any vanadium oxide species possesses a significant scattering path (displayed in green) attributable to the V=O bond which is not present in the experimental data. Additionally, all acyclic binding modes and both 1:1 cyclic binding modes possess insufficient scattering paths at 2.5 Å to adequately fit the feature in the experimental data. From these observations we can conclude the vanadium in seawater-contacted adsorbent polymers is a non-oxido species which is bound in 2:1 fashion by cyclic imide-dioxime groups.

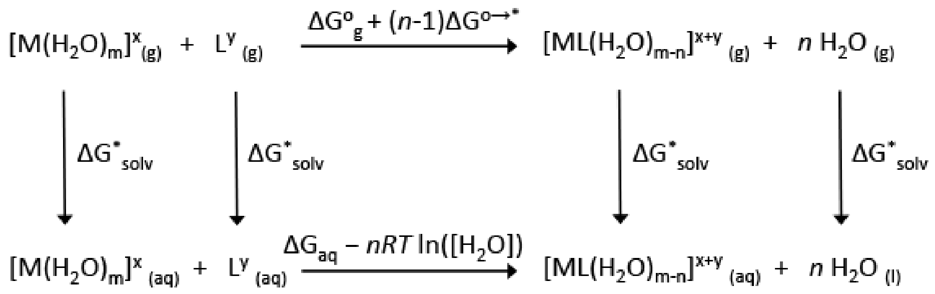

Supplementary Figure 9. Thermodynamic cycle used to calculate ΔG_{aq}.

Supplementary Table 1. Equilibrium constants for the reactions, $p\mathrm{H_2VO_4^-} + q\mathrm{H^+} + r\mathrm{HL^{2-}} \rightleftharpoons (\mathrm{H_2VO_4^-})_p(\mathrm{H^+})_q(\mathrm{HL^{2-}})_r$, at 25 °C and I = 0.6 M (NaCl), in the absence of ligands $HIDO^{2-}$ or AO^- (i.e., r = 0).

p,q,r	*Species*	*Reactions*	*log β_{pqr}**
1,-2,0	VO_4^{3-}	$H_2VO_4^- \rightleftharpoons VO_4^{3-} + 2H^+$	-21.31
1,-1,0	HVO_4^{2-}	$H_2VO_4^- \rightleftharpoons HVO_4^{2-} + H^+$	-7.946
1,0,0	$H_2VO_4^-$	/	0
1,2,0	VO_2^+	$H_2VO_4^- + 2H^+ \rightleftharpoons VO_2^+ + 2H_2O$	6.92
2,-2,0	$V_2O_7^{4-}$	$2H_2VO_4^- \rightleftharpoons V_2O_7^{4-} + 2H^+ + H_2O$	-15.23
2,-1,0	$HV_2O_7^{3-}$	$2H_2VO_4^- \rightleftharpoons HV_2O_7^{3-} + H^+ + H_2O$	-5.44
2,0,0	$H_2V_2O_7^{2-}$	$2H_2VO_4^- \rightleftharpoons H_2V_2O_7^{2-} + H_2O$	2.79
4,-2,0	$V_4O_{13}^{6-}$	$4H_2VO_4^- \rightleftharpoons V_4O_{13}^{6-} + 2H^+ + 3H_2O$	-8.60
4,-1,0	$HV_4O_{13}^{5-}$	$4H_2VO_4^- \rightleftharpoons HV_4O_{13}^{5-} + H^+ + 3H_2O$	0.13
4,0,0	$V_4O_{12}^{4-}$	$4H_2VO_4^- \rightleftharpoons V_4O_{12}^{4-} + 4H_2O$	9.89
5,0,0	$V_5O_{15}^{5-}$	$5H_2VO_4^- \rightleftharpoons V_5O_{15}^{5-} + 5H_2O$	12.16
6,0,0	$V_6O_{18}^{6-}$	$6H_2VO_4^- \rightleftharpoons V_6O_{18}^{6-} + 6H_2O$	13.9
10,4,0	$V_{10}O_{28}^{6-}$	$10H_2VO_4^- + 4H^+ \rightleftharpoons V_{10}O_{28}^{6-} + 12H_2O$	51.76
10,5,0	$HV_{10}O_{28}^{5-}$	$10H_2VO_4^- + 5H^+ \rightleftharpoons HV_{10}O_{28}^{5-} + 12H_2O$	57.83
10,6,0	$H_2V_{10}O_{28}^{4-}$	$10H_2VO_4^- + 6H^+ \rightleftharpoons H_2V_{10}O_{28}^{4-} + 12H_2O$	61.43
10,7,0	$H_3V_{10}O_{28}^{3-}$	$10H_2VO_4^- + 7H^+ \rightleftharpoons H_3V_{10}O_{28}^{3-} + 12H_2O$	62.64

*The equilibrium constants are taken from supplementary reference 1.

Supplementary Table 2. Protonation constants for glutaroimide-dioxime (H_3IDO) and acetamidoxime (HAO).

p,q,r	*Reaction*	*log β_{pqr}*	*Ref.**
0, 1, 1	$H^+ + HIDO^{2-} \rightleftharpoons H_2IDO^-$	12.27 ± 0.03	2
0, 2, 1	$2H^+ + HIDO^{2-} \rightleftharpoons H_3IDO$	23.15 ± 0.12	2
0, 3, 1	$3H^+ + HIDO^{2-} \rightleftharpoons H_4IDO^+$	25.67 ± 0.12	2
0, 1, 1	$H^+ + AO^- \rightleftharpoons HAO$	13.21	3
0, 2, 1	$2H^+ + AO^- \rightleftharpoons H_2AO^+$	18.99	3

*Protonation constants are reported at 25 °C, I = 0.5 M (NaCl) for glutaroimide-dioxime (H_3IDO)[2] and at 25 °C, I = 0 M (NaCl) for acetamidoxime (HAO)[3].

Supplementary Table 3. Free energy of the reactions, ΔG_{aq} (kcal/mol), assessed at the CCSD(T)/aug-cc-pvDZ//M06/SSC/6-311++G(d,p) level of theory and using the SMD solvation model.

Reactions	*ΔG_{aq} (kcal/mol)*
$[VO(OH)(IDO)]^- + [VO(OH)(IDO)]^- \leftrightarrows [V(IDO)_2]^- + H_2VO_4^-$	-3.08
$[VO(OH)(HIDO)] + [VO(OH)(HIDO)] \leftrightarrows [V(HIDO)_2]^+ + H_2VO_4^-$	8.74
$[VO(OH)(IDO)]^- + [VO(OH)(HIDO)] \leftrightarrows [V(IDO)(HIDO)] + H_2VO_4^-$	0.03
$[VO(OH)(IDO)]^- + [VO_2(IDO)]^{2-} \leftrightarrows [V(IDO)_2]^- + HVO_4^{2-}$	4.95
$3[VO_2(AO)(H_2O)] \leftrightarrows [V(AO)_3]^{2+} + 2H_2VO_4^- + H_2O$	18.51
$3[VO_2(AO)(H_2O)] \leftrightarrows [V(AO)(AO\_H)_2] + 2H_3VO_4 + H_2O$	6.11

Supplementary Table 4. Enthalpy of protonation and complexation reactions (I = 0.5 M NaCl for all data except those from 4 for I = 1 M $NaClO_4$).

p,q,r	*Reactions*	*ΔH, kJ mol^{-1}*	*ΔS, J mol^{-1} K^{-1}*	*Ref.**
1,-1,0	$H_2VO_4^- \leftrightarrows HVO_4^{2-} + H^+$	9 ± 2	-122 ± 9	4
1,2,0	$H_2VO_4^- + 2H^+ \leftrightarrows VO_2^+ + 2H_2O$	-33 ± 2	20 ± 7	4
0,1,1	$H^+ + HIDO^{2-} \leftrightarrows HIDO^-$	-36.1 ± 0.5	110 ± 2	2
0,2,1	$2H^+ + HIDO^{2-} \leftrightarrows H_3IDO$	-69.7 ± 0.9	202 ± 3	2
0,3,1	$3H^+ + HIDO^{2-} \leftrightarrows H_4IDO^+$	-77 ± 6	218 ± 14	2
1,1,1	$H_2VO_4^- + H^+ + HIDO^{2-} \leftrightarrows VO_2(IDO)^{2-} + 2H_2O$	-64 ± 3	189 ± 8	p.w.
1,2,2	$H_2VO_4^- + 2H^+ + 2HIDO^{2-} \leftrightarrows VO_2(HIDO)_2^{3-} + 2H_2O$	-246 ± 5	-137 ± 15	p.w.
1,4,2	$H_2VO_4^- + 4H^+ + 2HIDO^{2-} \leftrightarrows VO_2(H_2IDO)_2^- + 2H_2O$	-146 ± 3	529 ± 8	p.w.

*p.w. = present work.

Supplementary Table 5. Concentration of Metals in Field Test Seawater and as Extracted by Polymer Adsorbents Analyzed by XAFS.

Metal	Seawater (μg/kg)	AF-1 (g M / kg Adsorbent)	AI-8 (g M / kg Adsorbent)
V	1.48	5.8	7.0
U	2.84	3.1	2.8
Fe	2.20	0.8	4.0
Cu	0.54	0.7	0.5
Ni	0.56	0.4	0.4
Zn	2.10	0.9	0.6
Sr	---	0.1	< 0.1
Cr	0.18	< 0.1	< 0.1
Mn	1.20	< 0.1	0.9

Supplementary Table 6. Scattering Paths Used to Fit Na_3VO_4 EXAFS Data.

Path	Degeneracy	Initial Distance
**V→O**	4	1.691
**V→ O→O**	12	3.072

Supplementary Table 7. Scattering Paths Used to Fit $Na[VO_2(HIDO)]$ EXAFS Data.

Path	Degeneracy	Initial Distance
**V→O**	2	1.637
**V→N_{imide}**	3[a]	1.989
**V→N_{oxime}**	4[b]	2.898
**V→ O_{oxime}→N_{oxime}**	8	3.107

[a] Including V→N_{imide} (degeneracy: 1) and V→O_{oxime} (degeneracy: 2)

[b] Including V→N_{oxime} (degeneracy: 2) and V→C_{cycle} (degeneracy: 2)

Supplementary Table 8. Scattering Paths Used to Fit $Na[V(IDO)_2]$ EXAFS Data.

Path	Degeneracy	Initial Distance
**$V \rightarrow O_{oxime}$**	6[a.]	1.902 / 1.946
**$V \rightarrow N_{oxime}$**	8	2.891 / 2.999
**$V \rightarrow N_{oxime} \rightarrow O_{oxime}$**	16	3.105 / 3.167

[a] Including $V \rightarrow N_{imide}$ (degeneracy: 2) and $V \rightarrow O_{oxime}$ (degeneracy: 4)

Supplementary Table 9. Scattering Paths Used to Fit Seawater-Contacted Adsorbents.

Path	Degeneracy	Initial Distance[a.]
**$V \rightarrow O_{oxime}$**	4	1.946
**$V \rightarrow N_{imide}$**	2	2.157
**$V \rightarrow N_{oxime}$**	8	2.999
**$V \rightarrow N_{oxime} \rightarrow O_{oxime}$**	16	3.167

[a] Structure model for seawater-contacted adsorbents was lengthened from the crystal structure to account for polymer morphology influences

Supplementary Table 10. Refined Parameters for Fit of Na_3VO_4.

	Crystalline	Aqueous
**S_0^2**	0.75 ± 0.10	0.82 ± 0.15
**ΔE_0 (eV)**	-2.3 ± 2.0	-6.2 ± 2.8
**Δr_O (Å)**	0.01 ± 0.01	-0.009 ± 0.010
**σ^2_O (×10^{-3} Å^2)**	2.3 ± 0.8	2.1 ± 0.8

Supplementary Table 11. Refined Parameters for Fit of Na[VO_2(HIDO)].

	**Crystalline**	**Aqueous**
$\mathbf{S_0^2}$	0.98 ± 0.20	0.86 ± 0.15
$\mathbf{\Delta E_0}$ **(eV)**	-2.5 ± 3.0	-4.6 ± 3.5
$\mathbf{\Delta r_O}$ **(Å)**	0.03 ± 0.04	0.01 ± 0.03
$\mathbf{\sigma^2_O}$ **(×10^{-3} Å^2)**	3.5 ± 0.9	2.6 ± 0.9
$\mathbf{\Delta r_{Nimide}}$ **(Å)**	0.003 ± 0.03	-0.001 ± 0.02
$\mathbf{\sigma^2_N}$ **(×10^{-3} Å^2)**	4.5 ± 1.0	4.7 ± 1.5
$\mathbf{\Delta r_{Noxime}}$ **(Å)**	0.01 ± 0.05	-0.005 ± 0.05

Supplementary Table 12. Refined Parameters for Fit of Na[V(IDO)$_2$].

	**Crystalline**	**Aqueous**
$\mathbf{S_0^2}$	0.72 ± 0.15	0.74 ± 0.10
$\mathbf{\Delta E_0}$ **(eV)**	7.8 ± 4.3	7.4 ± 2.3
$\mathbf{\Delta r_{Ooxime}}$ **(Å)**	0.05 ± 0.02	0.01± 0.01
$\mathbf{\sigma^2_O}$ **(×10^{-3} Å^2)**	2.1 ± 0.9	2.3 ± 1.0
$\mathbf{\Delta r_{Noxime}}$ **(Å)**	0.02 ± 0.02	0.02 ± 0.02
$\mathbf{\sigma^2_N}$ **(×10^{-3} Å^2)**	2.9 ± 0.8	2.5± 0.8

Supplementary Table 13. Refined Parameters for Fit of Seawater-Contacted Adsorbents.

	**AF-1**	**AI-8**
$\mathbf{S_0^2}$	1.01 ± 0.07	0.83 ± 0.07
$\mathbf{\Delta E_0}$ **(eV)**	-1 ± 2	0 ± 2
$\mathbf{\Delta r_{Ooxime}}$ **(Å)**	-0.02 ± 0.01	-0.01 ± 0.01
$\mathbf{\sigma^2_O}$ **(×10^{-3} Å^2)**	2.0 ± 0.4	2.0 ± 0.4
$\mathbf{\Delta r_{Nimide}}$ **(Å)**	0.21 ± 0.02	0.23 ± 0.03
$\mathbf{\sigma^2_N}$ **(×10^{-3} Å^2)**	5 ± 2	4 ± 2
$\mathbf{\Delta r_{Noxime}}$ **(Å)**	-0.12 ± 0.01	-0.11 ± 0.2

Supplementary Table 14. Summary of Fit Metrics.

	**Na[VO_2(HIDO)] (Crystalline)**	**Na[VO_2(HIDO)] (Aqueous)**	**Na[V($IDO)_2$] (Crystalline)**	**Na[V($IDO)_2$] (Aqueous)**	**AF-1**	**AI-8**
**k-range**	2.50 – 10.2	2.50 – 10.2	2.75-10.2	2.75-10.2	2.75 – 10.2	2.75 – 10.2
**R-range**	1.0 – 3.5	1.0 – 3.5	1.1-3.5	1.1-3.5	1.1 – 3.5	1.1 – 3.5
**N_{IDP}**	11.9	11.9	11.3	11.3	11.3	11.3
**N_{var}**	7	7	6	6	7	7
**χ_v^2**	586	165	653	261	87.7	70.7
**R**	4.5%	3.3%	1.5%	1.1%	1.3%	1.7 %

Supplementary Table 15. Comparison of equilibrium constants for U(VI) and V(V) complexes with glutaroimide-dioxime (H_3IDO).

Reactions	*log β*	*Ref.*
$VO_2^+ + 2H^+ + 2HIDO^{2-} \leftrightarrows VO_2(H_2IDO)_2^-$	46.1 ± 0.4*	p.w.
$UO_2^{2+} + 2H^+ + 2HIDO^{2-} \leftrightarrows UO_2(H_2IDO)_2$	43.0 ± 1.1	2

*This value is obtained from the equilibrium constants for the reactions $H_2VO_4^- + 4H^+ + 2HIDO^{2-} \leftrightarrows VO_2(H_2IDO)_2^- + 2H_2O$ and $H_2VO_4^- + 2H^+ \leftrightarrows VO_2^+ + 2H_2O$.

Supplementary Table 16. Calculation of the enthalpy of reaction under seawater conditions.

Relevant Reactions	*ΔH, kJ mol⁻¹*	*Ref.*
$H_3IDO \leftrightarrows 2H^+ + HIDO^{2-}$	+(69.7 ± 0.9)	2
$H_2VO_4^- \leftrightarrows HVO_4^{2-} + H^+$	+(9 ± 2)	4
$H_2VO_4^- + 4H^+ + 2HIDO^{2-} \leftrightarrows V(IDO)_2^- + 4H_2O$	-(146 ± 3)	p.w.
**$H_2VO_4^- + 2H_3IDO \leftrightarrows V(IDO)_2^- + 4H_2O$**	**-(9 ± 5)**	**p.w.**
**$HVO_4^{-} + 2H_3 IDO + H^+ \leftrightarrows V(IDO)_2^- + 4H_2O$**	**-(18 ± 4)**	**p.w.**
**$Ca_2(UO_2)(CO_3)_3 + 2H_3IDO \leftrightarrows UO_2(H_2IDO)(HIDO)^- + 3HCO_3^- + Ca^{2+}$**	**+(30 ± 6)**	5

Supplementary Table 17. Crystallographic data of solid V(V) compounds for EXAFS studies.

<table>
<tr><th>Sample</th><th>Bond</th><th>N</th><th>R (Å)</th><th>Structure</th><th>Ref.</th></tr>
<tr><td>(a) $Na_3VO_{4(s)}$</td><td>V-O</td><td>4.0</td><td>1.691</td><td>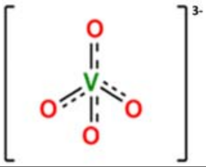</td><td>6</td></tr>
<tr><td rowspan="3">(b)
$Na[VO_2(HIDO)]_{(cr)}$</td><td>V-O</td><td>2.0</td><td>1.637 (V-O14)
1.678 (V-O3)</td><td rowspan="3">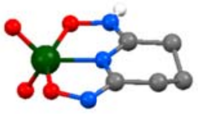</td><td rowspan="3">7</td></tr>
<tr><td>V-O/N</td><td>3.0</td><td>1.893 (V-O2)
2.005 (V-O5)
1.989 (V-N6)</td></tr>
<tr><td>V-N/C</td><td>4.0</td><td>2.883 (V-N1)
2.897 (V-C5)
2.890 (V-C9)
2.911 (V-N7)</td></tr>
<tr><td rowspan="2">(c) $Na[V(IDO)_2]_{(cr)}$</td><td>V-O/N</td><td>6.0</td><td>1.867 (V-O1)
1.903 (V-O2/3)
1.874 (V-O4)
1.956 (V-N14)</td><td rowspan="2">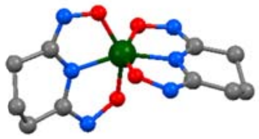</td><td rowspan="2">7</td></tr>
<tr><td>V-N/C</td><td>8.0</td><td>2.878 (V-N2)
2.884 (V-N5)
2.890 (V-N5)
2.870 (V-N6)
2.863 (V-C1)
2.864 (V-C5)
2.859 (V-C6)
2.846 (V-C10)</td></tr>
</table>

Supplementary Methods:

Electronic structure calculations. Quantum chemical calculations were performed with the Gaussian 09 D.01 software.[8] The density functional theory (DFT) approach was adopted using the M06[9] density functional with the standard Stuttgart small-core (SSC) 1997 relativistic effective core potential (RECP),[10] the associated contracted [6s/5p/3d/1f] basis set for vanadium and the 6-311++G(d,p) basis set for the light atoms. Frequency calculations were performed at the B3LYP/SSC/6-31+G(d)[11] level to ensure that geometries (optimized at the same B3LYP/SSC/6-31+G(d) level) were minima and to compute zero-point energies and thermal corrections. Using the gas-phase geometries, implicit solvent corrections were obtained at 298 K with the SMD[12] solvation model as implemented in Gaussian 09 at the B3LYP/SSC/6-31+G(d) level of theory.

The preference for using a combination of the M06 and the B3LYP functionals with the SMD solvation model was based on the results of our previous studies,[13,14] which showed that the chosen level of theory provides the best overall performance in predicting the log β values of uranyl[13] and vanadium[14] ions complexes with anionic oxygen and amidoxime donor ligands. Additionally, single-point coupled-cluster theory calculations, CCSD(T)/aug-cc-pvDZ (the valence electrons on C, O, H and the valence and subvalence electrons (3s, 3p) on V were correlated), using M06/SSC/6-311++G(d,p) optimized geometries were employed for selected V complexes with H_3IDO and HAO ligands.

Free energies and stability constants calculations. Complexation free energies in aqueous solution, ΔG_{aq}, and stability constants, log β^{theor}, were calculated using the methodology described in our previous work on V(V) and V(IV) containing complexes.[14] According to the thermodynamic cycle shown in Supplementary Figure 9, ΔG_{aq} is given by:

$$\Delta G_{aq} = \Delta G^{o}_{g} + \Delta\Delta G^{*}_{solv} + (n\text{-}1)\Delta G^{o\rightarrow *} + nRT\ln([H_2O]) \quad (1)$$

where ΔG^{o}_{g} is the free energy of complexation in the gas phase and $\Delta\Delta G^{*}_{solv}$ is the difference in the solvation free energies for a complexation reaction:

$$\Delta\Delta G^{*}_{solv} = \Delta G^{*}_{solv}([ML(H_2O)_{m\text{-}n}]^{x+y}) + n\Delta G^{*}_{solv}(H_2O) - \Delta G^{*}_{solv}([M(H_2O)_m]^{x}) - \Delta G^{*}_{solv}(L^{y}) \quad (2)$$

where L^y denotes the ligand with a charge of y and M is VO_2^+. The standard state correction terms must be introduced to connect ΔG^{o}_{g}, $\Delta\Delta G^{*}_{solv}$, and ΔG_{aq}, which are defined using different

standard state conventions. The free energy change for the conversion of 1 mol of solute from the gas phase at a standard state of 1 atm (24.46 L/mol) to the aqueous phase at a standard state of 1 mol/L at 298.15 K is given by $\Delta G^{o \rightarrow *}$ = 1.89 kcal/mol. Likewise, $RT \ln([H_2O])$ = 2.38 kcal/mol (T = 298.15 K) is the free energy change for the conversion of 1 mol of solvent from the aqueous phase at 1 mol/L to pure water at a standard state of 55.34 mol/L. Lastly, the stability constant (log β) value is related to free energy change for the complexation reaction by the following equation:

$$\log K_1 = \frac{-\Delta G_{aq}}{2.303 \cdot RT} \tag{3}$$

Since the formation of "bare" vanadium complexes is an extremely rare event, the developed protocol cannot be directly applied to determine log β of the non-oxido 1:2 and 1:3 V(V) species. However, the corresponding log β values can be obtained by a combination of the reactions for which ΔG_{aq} is experimentally known or was calculated at the CCSD(T) level.

Ab initio MD simulations. The ab initio MD simulations in the Helmholtz ensemble (NVT) were performed using the VASP (Vienna Ab initio Simulation Package), version 5.3.5.[15] The forces on the ions are calculated from the electronic structure of the system using DFT at the generalized gradient approximation level employing the PBE functional.[16] For the $[V(IDO)_2]^-$ complex: a simulation box of (19.45×12.77×12.40 Å^3) was used, containing the $[V(IDO)_2]^-$ complex, one Na^+ counterion to maintain the simulation box neutral, and 85 water molecules as solvent medium. For the $[V(AO)_3]^{2+}$ complex: a cubic simulation box of 13.04 Å was used, containing the $[V(AO)_3]^{2+}$ complex, two HO^- or two Cl^- counterions and 62 water molecules as solvent medium. The energy cutoff was set to 400 eV and given the large size of the unit cell the *k*-space representation included only the Γ point. The initial structure of each system was first minimized with 250 steps, and then the system was equilibrated for 5 ps at 300 K, followed by a 5 ps production run.

Chemicals. Freshly boiled/cooled Millipore water was used to prepare all solutions. Anhydrous sodium metavanadate ($NaVO_3$, 99.9%, Aldrich), sodium orthovanadate (Na_3VO_4, 99.9%, Aldrich), H_2SO_4 (70%, Sigma Aldrich), potassium hydrogen phthalate (KHP, 99.95-100.05%, Sigma Aldrich), tris(hydroxymethyl)aminomethane (TRIS, 99.9995%, Fluka), and ferrous ammonium sulfate hexahydrate (Baker and Adamson) were used as received. Glutaroimide-

dioxime (H_3IDO) was synthesized according to previously reported procedures.[2] Sodium chloride (J.T. Baker) was recrystallized from water and was used to adjust the total ionic strengths (I) of all solutions to 0.5 M, which is similar to the salinity of seawater. Acetamidoxime was synthesized by stirring a mixture of 50% aqueous hydroxylamine solution (6 mL), acetonitrile (6 mL), and ethanol (6 mL) at room temperature for 24 hours, followed by heating to 60°C until all the volatiles evaporated and a white solid remained. It was then dissolved in water (5 mL) and evaporated again. The resulting solid was redissolved in water and was confirmed to contain only acetamidoxime by ^1H and ^{13}C NMR. Its concentration was assayed by ^1H NMR and protonation titration, then diluted to appropriate concentrations for experiments. ^1H NMR (H_2O/D_2O, 600 MHz): δ 1.87 (3H). ^{13}C NMR (H_2O/D_2O, 151 MHz): δ 154.6 ($C(NH_2)(NOH)$), 14.9 (CH_3). Potassium dichromate was crushed and dried at 150 °C for two hours before use.

Stock solutions of sodium hydroxide titrant were prepared from a 1.00 M volumetric solution (Metrohm) and standardized against KHP. Hydrochloric acid stock solutions were prepared using a 1.00 M volumetric solution (Fluka) and subsequently standardized against tris(hydroxymethyl)aminomethane (TRIS, 99.9995%, Fluka). A stock solution of 0.0383 M $NaVO_3$ was prepared by dissolving the appropriate amount of $NaVO_3$ in water and was standardized against ferrous ammonium sulfate in 6 M H_2SO_4 using N-phenylanthranilic acid as the indicator.[17] The ferrous ammonium sulfate solution was standardized against potassium dichromate (primary standard) in 1 M H_2SO_4 using N-phenylanthranilic acid as the indicator.[18] Generally, the stock solutions used for all complexation titrations (potentiometric and calorimetric) were prepared by combining the appropriate masses of $NaVO_3$ stock solution, solid glutaroimide-dioxime, NaCl, and the appropriate volume of standard 0.1 M NaOH and diluting to volume with water. The densities of the solutions were then measured before allowing them to equilibrate overnight. Note that the pH of the stock solutions were adjusted to 11-12 because it was observed that such solutions remained stable for at least 7 days based on daily pH measurements ($\Delta pH < \pm 0.02$ after 7 days).

Potentiometric titrations. Potentiometric titrations were carried out using an automated titration system that consists of a thermostated glass vessel, Metrohm model 713 pH meter, and a Metrohm model 765 Dosimat. The temperature of the titration solutions in the glass vessel was maintained at 25.00 ± 0.01 °C using a circulating water bath. In order to minimize the ingress of CO_2, the solutions were blanketed with a gentle stream of argon. A Metrohm Unitrode

combination glass electrode was used to measure the electrode potentials in the titrations. Equations (1) and (2) describe the electrode potentials measured during a titration for the acidic and basic regions, respectively:

$$E = E^0 + 2.303 \times (RT/F) \log[H^+] + \gamma_H[H^+] \quad (4)$$

$$E = E^0 + 2.303 \times (RT/F) \log(Qw/[OH^-]) + \gamma_{OH}[OH^-] \quad (5)$$

E^0 is the standard electrode potential, $2.303 \times RT/F = 59.16$ mV for the hydrogen ion at 25 °C, $Qw = [H^+][OH^-]$, and $\gamma_H[H^+]$ and $\gamma_{OH}[OH^-]$ are the electrode junction potential corrections. Prior to each complexation titration, the electrode potentials measured from titrating a standard NaOH solution with a standard HCl solution were used to determine the parameters Qw, E^0, γ_H, and γ_{OH}. These values were then used to convert the measured potentials in the subsequent complexation titrations to the concentrations of H^+ for data analysis.

Five titrations were carried out to identify and determine the stability constants of V(V)-glutaroimide-dioxime complexes. The V(V) concentrations ranged from 1 to 5 mM and the glutaroimide-dioxime concentrations ranged from 3 mM to 20 mM. Typically, the metal to ligand ratio (M:L) was less than 1:2.5 to discourage formation of polynuclear hydrolysis species. For all complexation titrations, a standard 0.10 M HCl (I = 0.5 M) solution was used as the titrant. The titrations were carried out in a pH range of 2-12, and each titration consisted of at least 75 data points. A typical titration lasted at least 8 hours to ensure that equilibrium had been reached throughout the titration. The stability constants were calculated using the non-linear regression program Hyperquad 2008.[19] Multiple sets of titration data were refined in one input file. The hydrolysis constants for mononuclear and polynuclear vanadium species (e.g., $V_{10}O_{28}^{6-}$, $V_2O_7^{4-}$, etc.) reported by Pettersson et al.[1] (Supplementary Table 1) were included in the speciation model for the Hyperquad input file.

Calorimetric titrations. A TAM III microcalorimeter (TA instruments) was used to measure the enthalpies of complexation for V(V) complexes with H_3IDO. The titration assembly consists of two matching stainless steel vessels (1 mL total volume) as the reference and sample cells, a gold stirrer, and gas-tight syringes (0.250 mL total volume) equipped with either gold or stainless steel needles for titrant injections. During a given titration, the calorimeter measures the heat flow from the sample cell as the titrant is injected into the titration solution and compares it to that of the reference cell. Integrating the measured heat flow as a function of time for each injection interval generates the heat absorbed or released as a result of dilution and/or complexation. Prior

to each experiment, a dynamic electrical calibration was performed to determine the gain, offset, and time constants τ_1 and τ_2 for the calorimeter to calibrate the measured heat flow during titrations.

Four complexation titrations were carried out in which 0.75 mL 1 mM $NaVO_3$/4.1 mM H_3IDO/5 mM NaOH (I = 0.5 M) was titrated with 0.25 mL 0.040 M HCl (I = 0.5 M). Fifty 5 μL injections were added with six minutes between injections to determine the reaction heats, *Qr*, corresponding to each addition. The observed reaction heats were then corrected by subtracting the dilution heat, which was determined in separate dilution experiments, to obtain the net reaction heats that were used as input data in the program HypDeltaH[20] to calculate the enthalpies of complexation. The calculated V(V)-glutaroimide-dioxime complex stability constants, glutaroimide-dioxime protonation constants, V(V) hydrolysis constants, enthalpies of hydrolysis for mononuclear V(V) species, and the total reagent concentrations were also included as input data into HypDeltaH. The $H_2VO_4^-$ - H^+ - $HIDO^{2-}$ notation was also used in HypDeltaH to define V(V) complexes in the model. Notably, polynuclear vanadium species were not included because titration simulations using previously determined stability constants indicated that these species were minor and would not be significant contributors to the total heats under the titration conditions.

NMR studies. ^1H spectra were acquired on a Bruker AV-500 instrument (500 MHz), using a WATERGATE solvent suppression pulse sequence. Spectra were referenced to an external standard of C_6D_6. ^{51}V spectra were acquired on a Bruker DRX-500 instrument (132 MHz). Spectra were referenced to an external standard of dilute $VOCl_3$ in C_6D_6.

XAFS sample preparation. The mass of uranium needed to achieve a 1 – 2.5 absorption length edge step was calculated for each small molecule standard based on the elemental composition and mass absorption coefficient for each element. Na[VO_2(HIDO)], Na[V$(IDO)_2$], and vanadium oxide standards were ground with an agate mortar and pestle and blended with boron nitride to the appropriate concentration. For seawater contacted fibers, approximately 100 mg wet fibers were washed with DI water, dried overnight at 40 °C in a vacuum oven, immersed in liquid nitrogen and pulverized with a mortar and pestle. Samples contacted with the brine solution were diluted with D-(+)-Glucose to minimize self-absorption effects, while samples contacted with seawater were not diluted.

Small molecule and vanadium oxide standards were loaded on an aluminum holder with rectangular opening of 20 mm (*l*) x 2 mm (*w*) and a thickness of 0.5 – 1.0 mm, sealed with Kapton tape. For liquid samples, a Teflon holder was used to contain the samples, sealed with Kapton tape. Polymer samples were enclosed in a Nylon washer of 4.953 mm inner diameter, pressed into a self-supporting pellet, and sealed on both sides with Kapton tape. The entire sample was then placed into a baggie formed of Kapton film, which was then sealed with Kapton tape. This method of sample containment was approved by the APS Radiation Safety Review Board to ensure double containment for analysis of radioactive samples, as seawater-contacted adsorbents also contain appreciable quantities of natural uranium.

XAFS measurements. XAFS data were collected at the V K-edge (5465 eV) on beamline 10-BM-B of the Advanced Photon Source[21] for the polymers and vanadium oxide standards, and beamline 4-3 of the Stanford Synchrotron Radiation Lightsource for the liquid samples as well as $Na[VO_2(HIDO)]$ and $Na[V(IDO)_2]$ small molecule standards. XAFS spectra of the vanadium oxides and small molecule standards were collected in transmission mode, while the liquid samples and polymer samples were collected using a fluorescence detector. For beamline 10-BM-B, a Hitachi Vortex-ME4 four-element silicon drift fluorescence detector was used. For beamline 4-3, a Lytle or Vortex detector was used. For all samples, data sets were collected until adequate signal to noise was obtained, in all instances between 3 and 10 scans. The x-ray white beam was monochromatized by a Si(111) monochromator and detuned by 50% to reduce the contribution of higher-order harmonics. The K-edge of a vanadium foil was used as the reference for energy calibration and measured simultaneously for all samples. All samples were collected at room temperature.

XAFS data analysis. Data were processed with the Athena and Artemis programs of the IFEFFIT package.[22] Reference foil data were aligned to the first zero-crossing of the second derivative of normalized μ(E) data, which was calibrated to the literature E_0 value for the vanadium K-edge. Spectra were averaged in μ(E) prior to normalization. Background removal was achieved by spline fitting.

EXAFS data were extracted above the threshold energy, E_0. FEFF 9[23] was used to calculate theoretical phases and amplitudes from structure models consisting of crystal structures or the Cartesian coordinates of geometrically optimized computational models. All data were initially fitted with simultaneous k-weighting of 1,2, and 3, then finalized with k^2-weighting in R-space.

Fit windows in k-space were determined based on the lowest quality data collected, and for all data sets were from 2.75 – 10.2 Å^{-1}. Fit windows in R-space were determined on a case-by-case basis, based on the features apparent in the spectrum. In all fits, the amplitude reduction factor (S_0^2) and energy shift of the photoelectron (ΔE_0) were global parameters. Independent structural parameters determined by the fits included the change in the scattering half path length (ΔR_i) and the relative mean square displacement of the scattering element (σ^2_i). For each fit, the number of variables was not permitted to exceed 2/3 the number of independent points, in keeping with the Nyquist criterion.[24]

Synthesis of Na[VO_2(HIDO)]$_{(cr)}$ and Na[V$(IDO)_2$]$_{(cr)}$ for EXAFS studies. *Na[VO_2(HIDO)]$_{(cr)}$*. Sodium orthovanadate (184 mg, 1 mmol) was dissolved in 7 mL deionized water prior to adding glutaroimide-dioxime (143 mg, 1 mmol). The mixture was stirred at room temperature for 1 hour, during which the majority of the ligand dissolved and the solution became a bright orange color. The reaction mixture was filtered and then layered with an equal volume of isopropanol. After 3 days, the crystals that formed were decanted and dried under vacuum. The solids were checked by ^{1}H and ^{51}V NMR and were found to be identical to the previously reported Na[VO(OH)IDO].[7]

Na[V$(IDO)_2$]$_{(cr)}$: Sodium orthovanadate (244 mg, 2 mmol) was dissolved in 15 mL deionized water prior to adding glutaroimide-dioxime (572 mg, 4 mmol). The mixture was stirred for 1 hour and the color turned orange with a large amount of undissolved ligand present. Aqueous hydrochloric acid (approx. 3M) was added dropwise until a dark brown color persisted and the pH of the solution was between 6 and 7. The solution was then stirred for 1 hour at room temperature during which most of the remaining solid dissolved. The solution was filtered and allowed to evaporate to dryness. The solid residue was rinsed with a small amount of water at 0°C. The solid was checked by ^{1}H and ^{51}V NMR and was found to be identical to the previously reported Na[V$(IDO)_2$].[7]

Supplementary Note 1. Derivation of stability constants using ab initio calculations.

Complexation free energies in aqueous solution, ΔG_{aq}, and stability constants, $log\ \beta^{theor}$, were calculated using the methodology described in our previous work on V(V) and V(IV) containing complexes[14]. However, since the formation of "bare" vanadium complexes is an extremely rare event, the developed protocol cannot be directly applied to determine $log\ \beta^{theor}$ of the non-oxido 1:2 and 1:3 V(V) species. The corresponding $log\ \beta^{theor}$ values can be obtained by a combination of the reactions for which ΔG_{aq} is experimentally known or was calculated at the CCSD(T) level. For example, the complexation free energy, ΔG_{aq9}, for the formation of the non-oxido $[V(IDO)_2]^-$ complex was found by combination of the following reactions ($\Delta G_{aq9} = 2\Delta G_{aq6} + 2G_{aq7} + \Delta G_{aq8}$):

ΔG_{aq6} : $H_2VO_4^- + 2H^+ \leftrightarrows VO_2^+ + 2H_2O$ (6)

ΔG_{aq7} : $VO_2^+ + HIDO^{2-} \leftrightarrows [VO(OH)(IDO)]^-$ (7)

ΔG_{aq8} : $[VO(OH)(IDO)]^- + [VO(OH)(IDO)]^- \leftrightarrows [V(IDO)_2]^- + H_2VO_4^-$ (8)

ΔG_{aq9} ($\Delta G_{aq9} = 2\Delta G_{aq6} + 2G_{aq7} + \Delta G_{aq8}$):

$\mathbf{H_2VO_4^- + 4H^+ + 2HIDO^{2-} \rightleftharpoons [V(IDO)_2]^- + 4H_2O}$ (9)

where ΔG_{aq6} is experimentally known value from Supplementary Table 1 (at 25 °C and $I = 0$ M), ΔG_{aq7} was determined using our methodology from supplementary reference 14, and the free energy, ΔG_{aq8}, was determined using single-point coupled-cluster theory calculations, CCSD(T)/aug-cc-pvDZ (the valence electrons on C, O, H and the valence and subvalence electrons (3s, 3p) on V were correlated) at the M06/SSC/6-311++G(d,p) optimized geometries (see Supplementary Table 3 for the ΔG_{aq} of the reactions assessed by CCSD(T) method). Following the same analysis, the equilibrium constants (log β) of the subsequent protonation of the $[V(IDO)_2]^-$ complex can be estimated:

ΔG_{aq10} : $H_2VO_4^- + 2H^+ \leftrightarrows VO_2^+ + 2H_2O$ (10)

ΔG_{aq11} : $VO_2^+ + H^+ + HIDO^{2-} \leftrightarrows [VO(OH)(HIDO)]$ (11)

ΔG_{aq12} : $[VO(OH)(HIDO)] + [VO(OH)(HIDO)] \leftrightarrows [V(HIDO)_2]^+ + H_2VO_4^-$ (12)

ΔG_{aq13} ($\Delta G_{aq13} = 2\Delta G_{aq10} + 2G_{aq11} + \Delta G_{aq12}$):

$H_2VO_4^- + 6H^+ + 2HIDO^{2-} \rightleftharpoons [V(HIDO)_2]^+ + 4H_2O$ (13)

ΔG_{aq14} : $H_2VO_4^- + 2H^+ \leftrightarrows VO_2^+ + 2H_2O$ (14)

ΔG_{aq15} : $VO_2^+ + H^+ + HIDO^{2-} \leftrightarrows [VO_2(H_2IDO)]$ (15)

ΔG_{aq16} : $[VO(OH)(IDO)]^- + [VO(OH)(HIDO)] \leftrightarrows [V(IDO)(HIDO)] + H_2VO_4^-$ (16)

ΔG_{aq17} : $VO_2^+ + HIDO^{2-} \leftrightarrows [VO(OH)(IDO)]^-$ (17)

ΔG_{18} ($\Delta G_{aq18} = 2\Delta G_{aq14} + G_{aq15} + \Delta G_{aq16} + \Delta G_{aq17}$):

$H_2VO_4^- + 5H^+ + 2HIDO^{2-} \rightleftharpoons [V(IDO)(HIDO)] + 4H_2O$ (18)

To derive the stability constant for the $[VO_2(IDO)]^{2-}$ complex, we combine the following reactions:

ΔG_{aq19} : $H_2VO_4^- + 4H^+ + 2HIDO^{2-} \leftrightarrows [V(IDO)_2]^- + 4H_2O$ (19)

ΔG_{aq20} : $H_2VO_4^- + 2H^+ + HIDO^{2-} \leftrightarrows [VO(OH)(IDO)]^- + 2H_2O$ (20)

ΔG_{aq21} : $[VO(OH)(IDO)]^- + [VO_2(IDO)]^{2-} \leftrightarrows [V(IDO)_2]^- + HVO_4^{2-}$ (21)

ΔG_{aq22} : $HVO_4^{2-} + H^+ \leftrightarrows H_2VO_4^-$ (22)

ΔG_{aq23} ($\Delta G_{aq23} = \Delta G_{aq19} - G_{aq20} - \Delta G_{aq21} - \Delta G_{aq22}$):

$H_2VO_4^- + H^+ + HIDO^{2-} \rightleftharpoons [VO_2(IDO)]^{2-} + 2H_2O$ (23)

Similarly, we have assessed *log* β^{theor} for the formation of possible 1:3 non-oxido V(V) complexes with acetamidoxime ligand (AO):

ΔG_{aq24} : $H_2VO_4^- + 2H^+ + AO^- \leftrightarrows [VO_2(AO)] + 2H_2O$ (24)

ΔG_{aq25}: $3[VO_2(AO)(H_2O)] \leftrightarrows [V(AO)_3]^{2+} + 2H_2VO_4^- + H_2O$ (25)

ΔG_{aq26} ($\Delta G_{aq26} = 3\Delta G_{aq24} + \Delta G_{aq25}$):

$H_2VO_4^- + 6H^+ + 3AO^- \rightleftharpoons [V(AO)_3]^{2+} + 4H_2O$ (26)

ΔG_{aq27} : $H_2VO_4^- + 2H^+ + AO^- \leftrightarrows [VO_2(AO)] + 2H_2O$ (27)

ΔG_{aq28} : $H_2VO_4^- + H^+ \leftrightarrows H_3VO_4$ (28)

ΔG_{aq29} : $3[VO_2(AO)(H_2O)] \leftrightarrows [V(AO)(AO\_H)_2] + 2H_3VO_4 + H_2O$ (29)

ΔG_{aq30} ($\Delta G_{aq30} = 3\Delta G_{aq27} - 2\Delta G_{aq28} + \Delta G_{aq29}$):

$H_2VO_4^- + 4H^+ + 3AO^- \rightleftharpoons [V(AO)(AO\_H)_2] + 4H_2O$ (30)

Supplementary Note 2. Pseudo Jahn-Teller effect in the $[V(IDO)_2]^-$ complex.

Distortion from a symmetric configuration of $[V(IDO)_2]^-$ can become an important parameter that determines the strength and selectivity of ligand binding to vanadate. To provide more insight, ab initio molecular dynamics (AIMD) calculations were carried out on the $[V(IDO)_2]^-$ complex inside a periodic box containing 84 water solvent molecules and one Na^+ counterion. The system was equilibrated for 5 ps at 300 K, followed by a 5 ps production run (see Supplementary Methods). The results confirmed the stability of $[V(IDO)_2]^-$ with the AIMD trajectory showing a similar V(V) coordination environment with the average V-N and V-O bond distances of 1.98 Å and 1.90 Å, respectively, in excellent agreement with the XRD determined average V-N (1.96 Å) and V-O (1.89 Å) bond lengths.[7] Therefore, possible crystal packing forces in the reported $[V(IDO)_2]^-$ crystal structure[7] are not responsible for the deviation from an octahedral geometry, suggesting that the observed structural distortion originates from the electronic effects. Indeed, as pointed out by Bersuker et al.[25] the best approach to investigate molecular geometries should be based on how electrons control molecular configurations. The vibronic coupling theory,[25] which considers how electronic states and their changes influence nuclear configurations, has proven to be extremely useful in rationalizing molecular shapes. The pseudo Jahn–Teller effect (PJTE) resulting from electron-vibration (vibronic) coupling is the only source of instability and distortions of high-symmetry configurations of any polyatomic

system in nondegenerate states. In the following, we prove on the basis of the PJT theory that a distortion in an octahedral complex of vanadium(V) with the glutaroimide-dioxime ligands is due to the strong coupling between the unoccupied molecular orbitals (UMOs) and occupied molecular orbitals (OMOs) in the $[V(IDO)_2]^-$ system.

To elucidate the nature of instability of the octahedral $[V(IDO)_2]^-$ toward PJTE deformations, we performed calculations on a $[V(IDO')_2]^-$ cluster model (Supplementary Figure 2), in which alkyl groups of the glutaroimide-dioxime ligands (H_3IDO) are substituted by hydrogen atoms (H_3IDO'). Unlike $[V(IDO)_2]^-$, the complex $[V(IDO')_2]^-$ with truncated ligands can be forced to adopt a high symmetry (D_{2d} point group) configuration.

According to our results, the D_{2d} symmetric structure (Supplementary Figure 2) is not a minimum on the potential energy surface, but is only a second-order saddle point, with one doubly degenerate imaginary frequency mode [$\omega_{1,2}$ (e) = 33.37i cm^{-1}]. Geometry optimization along this mode leads to the distorted C_s complex in Supplementary Figure 2, which is structurally similar to the $[V(IDO)_2]^-$ complex with the glutaroimide-dioxime ligands. Thorough examination of molecular orbitals show that the distortion of the D_{2d} symmetric configuration of $[V(IDO')_2]^-$ into the C_s structure along the e mode can occur due to the vibronic coupling of HOMO (e) and LUMO (b_1). The product of their symmetries contains the symmetry of the imaginary mode (e):

$$e \times b_1 = e \tag{31}$$

Hence, the symmetry rule[26] for the PJT effect is satisfied as is the second condition:[26] the symmetry of the imaginary mode (e) of the D_{2d} structure corresponds to the real symmetric (a'') mode in the distorted C_s complex. Based on the nondegenerate second order perturbation theory,[27] the stabilization of the distorted structure with respect to the undistorted structure is proportional to the inverse of the energy gap between states which get coupled under the distortion, that is, $\Delta E_{stab} \sim 1/(E[UMO] - E[OMO])$. So, closer are the energies of the two electronic states that couple, larger is the gain in stabilization due to distortions. The predicted HOMO−LUMO gap is 3.70 eV at the M06/aug-cc-pvDZ level. The other three closest in energy OMO–UMO pairs [HOMO (e) – LUMO+1 (a_1), HOMO (e) – LUMO+2 (b_2), and HOMO-2 (a_2) – LUMO+4 (e)] satisfying symmetry conditions, have slightly larger energy gaps: 3.98, 4.00,

and 4.98 eV, respectively. A more accurate estimate of the HOMO–LUMO gap can be obtained using time-dependent DFT calculations. Musgrave et al.[28] demonstrated that TD-DFT calculations reliably predict the HOMO–LUMO gaps with several tested DFT methods, thus confirming that the excitation energy of the first singlet excited state is probably the most reasonable representation of the HOMO–LUMO gap in molecules. The TD-M06/aug-cc-pvDZ calculations for the D_{2d} complex predict the HOMO–LUMO gap value of 2.08 eV. Evidently, the 2.08 eV HOMO–LUMO gap is small enough to cause the PJT effect in the D_{2d} symmetric $[V(IDO')_2]^-$ complex.

It is also worth mentioning that according to Bersuker et al.[25] the driving force of the PJTE is mainly added covalence: "the PJTE distortion takes place when it results in energy gain by adding covalent bonding between some of the atoms by this distortion. Indeed, in the starting high-symmetry configuration, all the electronic states, ground and excited, are orthogonal by definition. By distortion, their orthogonality is lifted, and a nonzero overlap between these functions occurs. If for two near-neighbor atoms the ground state wave function pertains (mainly) to one of them, while the excited state wave function belongs (mainly) to the other one, their overlap by distortion contributes to an additional covalent bonding between them (that was absent in the high-symmetry configuration)." Thus, the $[V(IDO)_2]^-$ complex tends to adopt the highly distorted octahedral geometry that sustains stronger interactions between the glutaroimide-dioxime ligands and the bare vanadium(V) ion.

Supplementary Note 3. Experimental enthalpies of complexation for oxido and non-oxido V(V) complexes with glutaroimide-dioxime ligand (H_3IDO).

Supplementary Table 4 lists the enthalpies and entropies of formation for the three complexes identified by potentiometric titrations along with the protonation enthalpies for glutaroimide-dioxime (H_3IDO) and aqueous vanadium species reported in the literature.[1,2] The formation of each of the complexes is very favorable as evidenced by the very exothermic enthalpies. The relatively high uncertainty associated with the $[VO(IDO)_2]^{3-}$ complex probably stems from its low concentration as well as the possible ingression of carbon dioxide in solutions of high pH during the calorimetric titration.

Important insights into the observed selectivity for vanadium can be gained by comparing the stability constants for glutaroimide-dioxime complexes with V(V) and U(VI). The $[V(IDO)_2]^-$ complex has the same stoichiometry as the 1:2 U(VI)/glutaroimide-dioxime complex previously studied[2] so that their stability constants can be compared. As shown in Supplementary Table 5, the V(V) complex is more than three orders of magnitude stronger than the U(VI) complex.

The temperature of seawater plays an important role in determining the timing and location of deployment of a seawater extraction system. The temperature dependence on sorption of V(V) or U(VI) can be predicted largely based on the sign and value of the enthalpy of reaction corresponding to the sorption reaction that is expected to occur under seawater conditions. For the vanadium-glutaroimide-dioxime system, the predominant species of vanadium, the ligand, and the complex at seawater pH (around 8) are $H_2VO_4^-/HVO_4^{2-}$, H_3IDO, and $V(IDO)_2^-$, respectively. Therefore, the major overall reactions responsible for the sorption of vanadium could probably be represented by reactions 32 and 33 (Supplementary Table 6):

$$\mathbf{H_2VO_4^- + 2H_3IDO \leftrightarrows V(IDO)_2^- + 4H_2O} \quad (32)$$

$$\mathbf{HVO_4^{-} + 2H_3\ IDO + H^+ \leftrightarrows V(IDO)_2^- + 4H_2O} \quad (33)$$

By combining the protonation enthalpies for vanadium and glutaroimide-dioxime along with the enthalpy of formation of the non-oxido V(V) complex that is expected to predominate in seawater conditions (reactions 34, 35, and 36):

$$H_3IDO \leftrightarrows 2H^+ + HIDO^{2-} \quad (34)$$

$$H_2VO_4^- \leftrightarrows HVO_4^{2-} + H^+ \quad (35)$$

$$H_2VO_4^- + 4H^+ + 2HIDO^{2-} \leftrightarrows V(IDO)_2^- + 4H_2O \quad (36)$$

the enthalpies of the major reactions (reactions 32 and 33) are calculated to be –(9 ± 5) kJ mol^{-1} and –(18 ± 4) kJ mol^{-1} respectively, as shown in Supplementary Table 6. Without taking into account other macroscopic factors that can also affect sorption of cations to polymeric sorbents, this result would imply that in a real seawater system, as the seawater temperature increases, the sorption of vanadium to polymeric sorbents containing the glutaroimide-dioxime moiety should decrease. On the other hand, for uranium, the large positive enthalpy of the major reaction 37:

$$\mathbf{Ca_2(UO_2)(CO_3)_3 + 2H_3IDO \leftrightarrows UO_2(H_2IDO)(HIDO)^- + 3HCO_3^- + Ca^{2+}} \quad (37)$$

should lead to the opposite effect. Noting that the magnitude of enthalpy of reaction 36 is larger than those of reactions 32 and 33 (Supplementary Table 6), it is expected that deployment of poly(acrylamidoxime) sorbents at higher temperatures may enhance the extraction of uranium compared to the extraction of vanadium.

Supplementary Note 4. Experimental study of the interactions between V(V) and acetamidoxime ligand (HAO).

To confirm the lack of binding between V(V) and acetamidoxime (HAO), ^{1}H and ^{51}V NMR experiments were performed on this system. The main observation from this data (Supplementary Figure 6) is that no interaction between the two species was observed under the experimental conditions: (1) pH 7.5 – 10 with $[V]_{total}$ = 7.5 mM, [HAO] = 15 mM (HAO/V ratio = 2); and (2) pH 8.5 with $[V]_{total}$ = 7.5 mM, [HAO] = 45 mM (HAO/V ratio = 6). All the signals in the ^{51}V spectra can be assigned to known vanadate species (vanadate exists as a complex mixture of oxoanions in aqueous solution dependent on concentration and pH; VO_4^{3-}, HVO_4^{2-}, $H_2VO_4^-$, $V_2O_7^{4-}$, $V_4O_{12}^{4-}$ were all observed in this experiment),[29] and no changes are observed when acetamidoxime was added. This is consistent with the results of ^{51}V NMR studies of the branch-chained glutardiamidoxime ligand, showing no interactions with V(V) in aqueous medium.[30] Additionally, no changes in chemical shift and no new signals were observed in the ^{1}H spectra of the acetamidoxime. To adjust the pH, 1 equivalent (with respect to vanadium) of HCl or NaOH was added to the ligand solution before adding the vanadate.

Supplementary References

1. Pettersson, L. & Elvingson, K. in *Vanadium Compounds*, **711**, 30–50, (1998).

2. Tian, G., Teat, S. J., Zhang, Z. & Rao, L. Sequestering uranium from seawater: binding strength and modes of uranyl complexes with glutarimidedioxime. *Dalton Trans.* **41**, 11579-11586, (2012).

3. Mehio, N. et al. Acidity of the amidoxime functional group in aqueous solution: a combined experimental and computational study. *J. Phys. Chem. B* **119**, 3567-3576, (2015).

4. Cruywagen, J. J., Heyns, J. B. B. & Westra, A. N. Protonation equilibria of mononuclear vanadate: thermodynamic evidence for the expansion of the coordination number in VO_2^+. *Inorg. Chem*. **35**, 1556–1559, (1996).

5. Leggett, C. J., Endrizzi, F. & Rao, L. Scientific basis for efficient extraction of uranium from seawater, II: fundamental thermodynamic and structural studies. *Ind. Eng. Chem. Res.*, **55**, 4257–4263, (2016).

6. Tillmanns, E. & Baur, W. H. On the crystal chemistry of salt hydrates. VII. The crystal structures of pseudo trisodium orthoarsenate dodecahydrate and the isomorphous phosphate and vanadate salts. *Acta Crystallogr. Sect. B* **27**, 2124–2132, (1971).

7. Leggett, C. J. et al. Structural and spectroscopic studies of a rare non-oxido V(V) complex crystallized from aqueous solution. *Chem. Sci.* **7**, 2775-2786, (2016).

8. Frisch, M. J. et al. Gaussian 09; Gaussian, Inc.: Wallingford CT, (2009).

9. Zhao, Y. & Truhlar, D. G. The M06 suite of density functionals for main group thermochemistry, thermochemical kinetics, noncovalent interactions, excited states, and transition elements: two new functionals and systematic testing of four M06-class functionals and 12 other functionals. *Theor. Chem. Acc.* **120**, 215-241, (2008).

10. Dolg, M., Stoll, H., Preuss, H. & Pitzer, R. M. Relativistic and correlation effects for element 105 (Hahnium, Ha). A comparative study of M and MO (M= Nb, Ta, Ha) using energy-adjusted ab initio pseudopotentials. *J. Phys. Chem*. **97**, 5852, (1993).

11. Becke, A. D. Density-functional thermochemistry. III. The role of exact exchange *J. Chem. Phys.* **98**, 5648-5652, (1993).

12. Marenich, A. V., Cramer, C. J. & Truhlar, D. G. J. Universal solvation model based on solute electron density and on a continuum model of the solvent defined by the bulk dielectric constant and atomic surface tensions. *Phys. Chem. B* **113**, 6378, (2009).

13. Mehio, N. et al. Quantifying the binding strength of salicylaldoxime–uranyl complexes relative to competing salicylaldoxime–transition metal ion complexes in aqueous solution: a combined experimental and computational study. *Dalton Trans.* **45**, 9051-9064, (2016).

14. Ivanov, A. S. & Bryantsev, V. S. Assessing ligand selectivity for uranium over vanadium ions to aid in the discovery of superior adsorbents for extraction of UO_2^{2+} from seawater. *Dalton Trans.* **45**, 10744-10751, (2016).

15. Kresse, G. & Hafner, J. Ab initio molecular dynamics for liquid metals. *Phys. Rev. B* **47**, 558-561, (1993).

16. Perdew, J. P., Burke, K. & Ernzerhof, M. Generalized gradient approximation made simple. *Phys. Rev. Lett.* **77**, 3865-3868, (1996).

17. Syrokomsky, W. S. & Stiepin, V. V. New oxidation-reduction indicators. I. Phenylanthranilic acid (o-diphenylamine carbonic acid). *J. Am. Chem. Soc*. **58**, 928–929, (1936).

18. Sriramam, K. Some observations on the redox behaviour of N-phenylanthranilic acid indicator in iron (II) titrations. *Talanta*, **20**, 383-390, (1973).
19. Alderighi, L. et al. Hyperquad Simulation and Speciation (HySS): a utility program for the investigation of equilibria involving soluble and partially soluble species. *Coord. Chem. Rev.* **184**, 311-318, (1999).
20. Gans, P., Sabatini, A. & Vacca, A. Simultaneous calculation of equilibrium constants and standard formation enthalpies from calorimetric data for systems with multiple equilibria in solution. *J. Solution Chem.* **37**, 467–476, (2008).

21. Kropf, A. J. et al. *AIP Conf. Proc.*, **1234**, 299-302, (2010).

22. Ravel, B. & Newville, M. ATHENA, ARTEMIS, HEPHAESTUS: data analysis for X-ray absorption spectroscopy using IFEFFIT. *J. Synchrotron Rad.* **12**, 537-541, (2005).

23. Rehr, J. J., et al. Parameter-free calculations of X-ray spectra with FEFF9. *Phys. Chem. Chem. Phys.* **12**, 5503-5513, (2010).

24. Calvin, S. in *XAFS for Everyone*. CRC press, (2013).
25. Bersuker, I. B. Pseudo-Jahn–Teller effect: a two-state paradigm in formation, deformation, and transformation of molecular systems and solids. *Chem. Rev.* **113**, 1351-1390, (2013).
26. Pearson, R. G. Concerning Jahn-Teller effects. *Proc. Natl. Acad. Sci. U. S. A.* **72**, 2104-2106, (1975).

27. Atkins, P. & Friedman, R. in *Molecular Quantum Mechanics*, 4th ed.; Oxford Univ. Press Inc.: NY, (2005).

28. Zhang, G. & Musgrave, C. B. Comparison of DFT methods for molecular orbital eigenvalue calculations *J. Phys. Chem. A* **111**, 1554–1561, (2007).

29. Howarth, O. W. Vanadium-51 NMR. *Progress in NMR Spectroscopy*, **22**, 453-485, (1990).

30. Pan, H. B. et al. Elution of uranium and transition metals from amidoxime-based polymer adsorbents for sequestering uranium from seawater. *Ind. Eng. Chem. Res.* **55**, 4313-4320, (2016).
